# *nab*-Paclitaxel/Carboplatin in Vulnerable Populations With Advanced Non-Small Cell Lung Cancer: Pooled Analysis

**DOI:** 10.3389/fonc.2020.485587

**Published:** 2021-01-26

**Authors:** Corey J. Langer, Ajeet Gajra, Cesare Gridelli, Kartik Konduri, Daniel Morgensztern, David Spigel, Denis Talbot, Michael Thomas, Jared Weiss, Richard Pilot, Rafia Bhore, Marianne Wolfsteiner, Teng Jin Ong, Mark Socinski

**Affiliations:** ^1^Abramson Cancer Center, University of Pennsylvania, Philadelphia, PA, United States; ^2^SUNY Upstate Medical University, Department of Medicine, Syracuse, NY, United States; ^3^S.G. Moscati Hospital, Division of Medical Oncology, Avellino, Italy; ^4^Baylor Charles A. Sammons Cancer Center, Texas Oncology PA, Dallas, TX, United States; ^5^Washington University School of Medicine in St Louis, St Louis, MO, United States; ^6^Sarah Cannon Research Institute, Lung Cancer Research Program, Nashville, TN, United States; ^7^Churchill Hospital—Oxford University Hospitals, Oxford, United Kingdom; ^8^Internistische Onkologie der Thoraxtumoren, Thoraxklinik im Universitätsklinikum Heidelberg, Translational Lung Research Center Heidelberg (TLRC-H), Heidelberg, Germany; ^9^Lineberger Comprehensive Cancer Center, University of North Carolina, Chapel Hill, NC, United States; ^10^Bristol Myers Squibb, Princeton, NJ, United States; ^11^PRA Health Sciences, Lenexa, KS, United States; ^12^AdventHealth Cancer Institute, Thoracic Cancer, Orlando, FL, United States

**Keywords:** chemotherapy, advanced non-small cell lung cancer, *nab*-Paclitaxel, platinum-based therapy, vulnerable populations

## Abstract

**Introduction:**

Despite improvements in the treatment of advanced non-small cell lung cancer (NSCLC), certain patient populations remain underrepresented in clinical trials. Many patients have benefited from platinum doublets, including *nab*-paclitaxel–based regimens, but there are patients with comorbidities who particularly require careful balancing of efficacy and safety. Clinical trial data are limited for patients who are elderly or have renal impairment, diabetes, or impaired performance status.

**Methods:**

To better understand outcomes in these patient populations, we performed a pooled analysis using data from the ABOUND clinical trial program (ABOUND.SQM, ABOUND.PS2, ABOUND.70+) and the key phase III trial of *nab*-paclitaxel/carboplatin in advanced NSCLC. The populations included in this pooled analysis consisted of elderly patients (≥ 70 years) and patients with renal impairment (eGFR < 60 ml/min/1.73 m^2^), diabetes, or poor performance status (ECOG PS 2).

**Results:**

Median progression-free survival (PFS) ranged from 4.1 months in patients with ECOG PS 2 (95% CI, 2.04–5.09 months) to 7.7 months in patients with diabetes (95% CI, 5.88–10.12 months). PFS for elderly patients and patients with renal impairment was 6.9 months each (95% CI, 6.01–7.98 months and 4.47–9.79 months, respectively). Median overall survival (OS) was 18.2 months (95% CI, 10.94–28.22 months), 17.4 months (95% CI, 14.59–20.14 months), and 16.1 months (95% CI, 14.09–18.50 months) in patients with renal impairment, patients with diabetes, and elderly patients, respectively. Patients with ECOG PS 2 exhibited the shortest median OS: 5.6 months (95% CI, 3.98–11.37 months). Overall response rates were 56.9%, 54.6%, 45.9%, and 29.4% in patients with diabetes, elderly patients, patients with renal impairment, and patients with ECOG PS 2, respectively. Most treatment-related adverse events were hematologic. The most common grade 3/4 hematologic adverse events in patients with renal impairment, elderly patients, patients with diabetes, and patients with poor performance status included neutropenia, anemia, and thrombocytopenia.

**Conclusions:**

Although survival data in patients with ECOG PS 2 were notably inferior to the other cohorts, our findings are consistent with those previously reported in the population-specific studies of the ABOUND trials and lend additional support for the use of *nab*-paclitaxel–based regimens in historically understudied and vulnerable populations.

## Introduction

Treatment of advanced non-small cell lung cancer (NSCLC) has greatly improved over the past 2 decades, and the recent advances with immunotherapy in advanced NSCLC are particularly encouraging (1, 2). However, some patient groups, including the elderly and patients with renal impairment, diabetes, or poor performance status, present unique treatment challenges because of comorbid conditions, perceptions of increased toxicity, short life expectancy, or compromised treatment efficacy, and underrepresentation in clinical trials (3–5). In addition, most clinical trials in advanced cancers stipulate normal renal function as an inclusion criterion, and elderly patients have historically been either excluded from or underrepresented in clinical trials despite accounting for the majority of lung cancer cases (6, 7). Immunotherapy has demonstrated benefit in patients with advanced NSCLC but has not yet been fully studied in many of these vulnerable populations (8). Although immunotherapy alone or in combination with chemotherapy is now a standard of care in a significant proportion of patients with advanced NSCLC, cytotoxic chemotherapy in the first and subsequent lines of therapy remains relevant.

Platinum-based combination chemotherapy, including *nab*-paclitaxel/carboplatin, has been shown to benefit many patient subgroups and was the recommended first-line treatment strategy for most patients prior to the advent of immunotherapy (9–11). Subset analyses of a key phase III trial indicated that treatment with *nab*-paclitaxel/carboplatin was associated with a clinical benefit and tolerable safety profile in various advanced NSCLC populations (12–14). The ABOUND clinical trial program assessed the role of *nab*-paclitaxel–based regimens in patients with squamous histology and also enrolled many patients who fell into these underserved subset categories. The ABOUND.70+ and ABOUND.PS2 studies highlighted the benefit and tolerability of *nab*-paclitaxel/carboplatin in elderly patients (≥ 70 years) and in patients with Eastern Cooperative Oncology Group performance status (ECOG PS) 2, respectively (15, 16).

The goal of this pooled analysis was to evaluate outcomes associated with *nab*-paclitaxel/carboplatin treatment of NSCLC in more vulnerable populations including the elderly and in patients with renal impairment, diabetes, or ECOG PS 2.

## Materials and Methods

This pooled analysis of ABOUND.SQM, ABOUND.PS2, ABOUND.70+, and the phase III study by Socinski et al. analyzed vulnerable populations with advanced NSCLC (**Supplemental Tables 1** **and** **2**; D Spigel, Unpublished data, 2019) (15–17). Study designs, methods, dosing, and schedule details have been previously published and are summarized in **Supplemental Table 3**. Treatment arms containing *nab*-paclitaxel/carboplatin were pooled across these four studies (patients from the ABOUND.SQM trial who received induction therapy with *nab*-paclitaxel/carboplatin followed by maintenance with best supportive care were excluded from this pooled analysis). In each of these studies and at each participating site, the studies were approved by IRB and the patients/participants provided written informed consent to participate in the studies.

Demographics and baseline patient characteristics were summarized in terms of frequencies or descriptive statistics for categorical or continuous data, respectively. The Kaplan-Meier product-limit method was used to estimate progression-free survival (PFS) and overall survival (OS) curves for each vulnerable population; median PFS and OS were defined as the shortest survival time in months in which estimated survival probability was ≤ 0.5. The Brookmeyer-Crowley method was used to estimate 95% CIs for medians. Median PFS and OS in the vulnerable population compared with those of the overall population of the pooled studies are presented in forest plots. No statistical comparisons were performed due to the heterogeneity of the pooled populations.

### Population Definitions

The patients included in this pooled analysis were not restricted to a single analytic cohort. For example, patients were placed in the poor performance status group if they had ECOG PS 2; however, this did not exclude them from other groups, if they qualified.

*Renal Impairment*: Renal impairment was defined by estimated glomerular filtration rate (eGFR; ml/min/1.73 m^2^). Moderate renal impairment included patients with an eGFR from ≥ 30 to < 60 ml/min/1.73 m^2^. Severe renal impairment included patients with an eGFR from ≥ 15 to < 30 ml/min/1.73 m^2^.

*Elderly Patients*: Elderly patients were defined as those 70 years of age or older. Age cutoff for elderly patients was chosen for the ABOUND.70+ trial and the phase III trial subgroup analysis and was maintained for this pooled analysis.

*Patients With Diabetes*: Patients with diabetes included those classified at baseline by the preferred terms “type 2 diabetes mellitus,” “diabetes mellitus,” and “glucose tolerance impaired” within the system organ class “metabolism and nutrition disorders.”

*Poor Performance Status*: Poor performance status was defined as ECOG PS 2.

## Results

### Patients

A total of 840 patients in this pooled population were analyzed. Of these, 66 (7.9%) had moderate or severe renal impairment, 293 (34.9%) were ≥ 70 years of age, 110 (13.1%) had a diagnosis of diabetes (including 7 patients classified as glucose tolerance impaired), and 42 (5.0%) had ECOG PS 2. Baseline characteristics were generally similar across these patient populations although weight and body mass index were highest in patients with diabetes and renal impairment (**Table 1**). The median age for each population ranged from 66.0 to 74.0 years. Patients were predominantly male, although gender was more evenly balanced among patients with renal impairment. Smoking status was similar across most populations, although patients with renal impairment included the highest proportion of never smokers (15.2%).

**Table 1:** Baseline characteristics.

Characteristic	Renal Impairment(n = 66)	Elderly(n = 293)	Diabetes^a^(n = 110)	Poor PS(n = 42)
**Age, median (range), years**	72.5	74.0	71.0	66.0
	(45.0-85.0)	(70.0–93.0)	(50.0–89.0)	(44.0–84.0)
**Age group, years, n (%)**				
< 65	16 (24.2)	0	30 (27.3)	19 (45.2)
≥ 65	50 (75.8)	293 (100.0)	80 (72.7)	23 (54.8)
< 70	23 (34.8)	0	49 (44.5)	24 (57.1)
≥ 70	43 (65.2)	293 (100.0)	61 (55.5)	18 (42.9)
< 75	39 (59.1)	148 (50.5)	75 (68.2)	31 (73.8)
≥ 75	27 (40.9)	145 (49.5)	35 (31.8)	11 (26.2)
**Gender, n (%)**				
Male	32 (48.5)	184 (62.8)	78 (70.9)	26 (61.9)
Female	34 (51.5)	109 (37.2)	32 (29.1)	16 (38.1)
**Weight, median (range), kg**	76.3	69.0^b^	80.0	70.1
	(36.2–120.2)	(36.2–117.2)	(46.0–124.7)	(42.9–111.1)
**Body mass index, median (range), kg/m^2^**	27.6	24.9^c^	27.7	24.4
	(17.5–47.0)	(16.0–40.5)	(17.6–41.5)	(15.7–41.5)
**Race, n (%)**				
Asian	1 (1.5)	18 (6.1)	12 (10.9)	0
African heritage	6 (9.1)	16 (5.5)	10 (9.1)	3 (7.1)
White	58 (87.9)	249 (85.0)	86 (78.2)	39 (92.9)
American Indian or Alaska native	0	2 (0.7)	0	0
Other	1 (1.5)	3 (1.0)	0	0
Unknown	0	5 (1.7)	2 (1.8)	0
**ECOG PS, n (%)**				
0	18 (27.3)	86 (29.4)	30 (27.3)	0
1	42 (63.6)	189 (64.5)	67 (60.9)	0
2	6 (9.1)	18 (6.1)	13 (11.8)	42 (100.0)
**Histology, n (%)**				
Adenocarcinoma	17 (25.8)	33 (11.3)	21 (19.1)	1 (2.4)
Squamous cell carcinoma	14 (21.2)	99 (33.8)	24 (21.8)	14 (33.3)
Non-squamous cell carcinoma	23 (34.8)	98 (33.4)	36 (32.7)	25 (59.5)
Other	3 (4.5)	6 (2.0)	1 (0.9)	1 (2.4)
Unknown	9 (13.6)	57 (19.5)	28 (25.5)	1 (2.4)
**Stage, n (%)**				
I	1 (1.5)	1 (0.3)	2 (1.8)	0
II	0	3 (1.0)	2 (1.8)	0
IIIA	2 (3.0)	12 (4.1)	5 (4.5)	0
IIIB	2 (3.0)	37 (12.6)	15 (13.6)	2 (4.8)
IV	60 (90.9)	236 (80.5)	86 (78.2)	40 (95.2)
Unknown	1 (1.5)	4 (1.4)	0	0
**Prior therapy, n (%)**				
Radiotherapy	10 (15.2)	49 (16.7)	23 (20.9)	12 (28.6)
Chemotherapy	0	2 (0.7)	3 (2.7)	0
Radiotherapy and chemotherapy	0	3 (1.0)	1 (0.9)	0
Unknown	56 (84.8)	239 (81.6)	83 (75.5)	30 (71.4)
**Smoking status, n (%)**				
Never smoked	10 (15.2)	18 (6.1)	6 (5.5)	0
Quit smoking	11 (16.7)	35 (11.9)	21 (19.1)	1 (2.4)
Currently smoking	7 (10.6)	21 (7.2)	4 (3.6)	2 (4.8)
Unknown	38 (57.6)	219 (74.7)	79 (71.8)	39 (92.9)
**Country, n (%)**				
Australia	0	1 (0.3)	0	0
Canada	2 (3.0)	4 (1.4)	1 (0.9)	1 (2.4)
Germany	2 (3.0)	7 (2.4)	6 (5.5)	0
Italy	0	3 (1.0)	0	0
Japan	1 (1.5)	15 (5.1)	9 (8.2)	0
Russia	11 (16.7)	17 (5.8)	9 (8.2)	0
Spain	1 (1.5)	3 (1.0)	3 (2.7)	0
Ukraine	6 (9.1)	13 (4.4)	1 (0.9)	0
United States	43 (65.2)	230 (78.5)	81 (73.6)	41 (97.6)
**Metformin therapy, n (%)**				
Yes	7 (10.6)	37 (12.6)	59 (53.6)	7 (16.7)
No	59 (89.4)	256 (87.4)	51 (46.4)	35 (83.3)
**Peripheral neuropathy at baseline, n (%)**				
No peripheral neuropathy	49 (74.2)	233 (79.5)	71 (64.5)	4 (9.5)
Grade 1	10 (15.2)	35 (11.9)	21 (19.1)	0
Grade 2	0	2 (0.7)	1 (0.9)	0
Unknown	7 (10.6)	23 (7.8)	17 (15.5)	38 (90.5)
**Renal impairment, eGFR ml/min/1.73 m^2^, n (%)**				
Moderate (eGFR ≥ 30–< 60)	65 (98.5)	42 (14.3)	14 (12.7)	6 (14.3)
Severe (eGFR ≥ 15–< 30)	1 (1.5)	1 (0.3)	0	0

ECOG PS, Eastern Oncology Cooperative Group performance status; eGFR, estimated glomerular filtration rate. ^a^ Includes seven patients classified as “glucose tolerance impaired.” ^b^ For weight of elderly population, n = 292. ^c^ For body mass index of elderly population, n = 291.

Overall, the treatment discontinuation rate was 97% among patients with renal impairment, patients aged ≥ 70 years, and patients with diabetes and 100% among patients with ECOG PS 2. Patients with ECOG PS 2 had a lower rate of discontinuation due to progressive disease (31.0% vs 39.3% to 43.9% in the other populations) but higher rates of discontinuation due to death (7.1% vs 1.5% to 3.1%), adverse events (33.3% vs 15.9% to 17.1%), and symptomatic deterioration (11.9% vs 3.0% to 6.3%) compared with patients in the other cohorts.

### Efficacy

#### Progression-Free Survival

**Figure 1:**
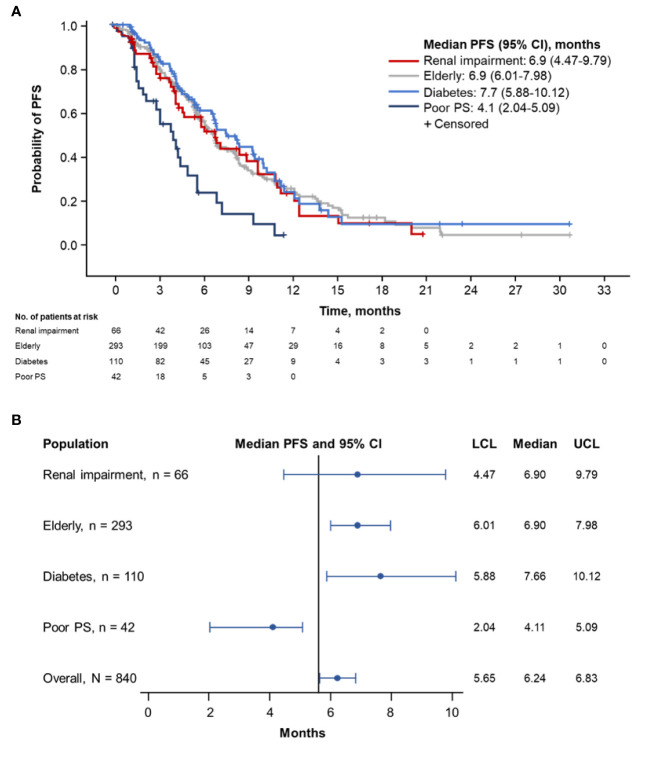
Kaplan-Meier curve **(A)** and forest plot **(B)** of PFS by population. PFS, progression-free survival; PS, performance status; LCL, lower confidence limit; UCL, upper confidence limit.

Patients with diabetes had the longest median PFS (7.7 months; 95% CI, 5.88–10.12 months), followed by elderly patients (6.9 months; 95% CI, 6.01–7.98 months) and patients with renal impairment (6.9 months; 95% CI, 4.47–9.79) (**Figures 1A, B****)**. Patients with ECOG PS 2 demonstrated the shortest median PFS (4.1 months; 95% CI, 2.04–5.09 months).

Designated subsets of the pooled populations underwent further comparative PFS analyses. No meaningful difference in median PFS was observed in patients with (n = 66) vs without (n = 774) renal impairment (6.9 months [95% CI, 4.47–9.79 months] vs 6.2 months [95% CI, 5.62–6.77 months]; **Supplemental Figure 1A**). The median PFS was similar in patients aged ≥ 70 years (n = 293) vs those aged < 70 years (n = 547) (6.9 months [95% CI, 6.01–7.98 months] vs 5.7 months [95% CI, 5.49–6.67 months]; **Supplemental Figure 1B**). No meaningful difference in median PFS was noted between patients with diabetes (n = 110) and those without diabetes (n = 730) (7.7 months [95% CI, 5.88–10.12 months] vs 6.0 months [95% CI, 5.55–6.57]; **Supplemental Figure 1C**) or in patients with diabetes who received metformin (n = 59) vs those who did not (n = 51) (6.8 months [95% CI, 4.40–10.38 months] vs 8.4 months [95% CI, 6.83–10.15 months]; **Supplemental Figure 1D**).

#### Overall Survival

**Figure 2:**
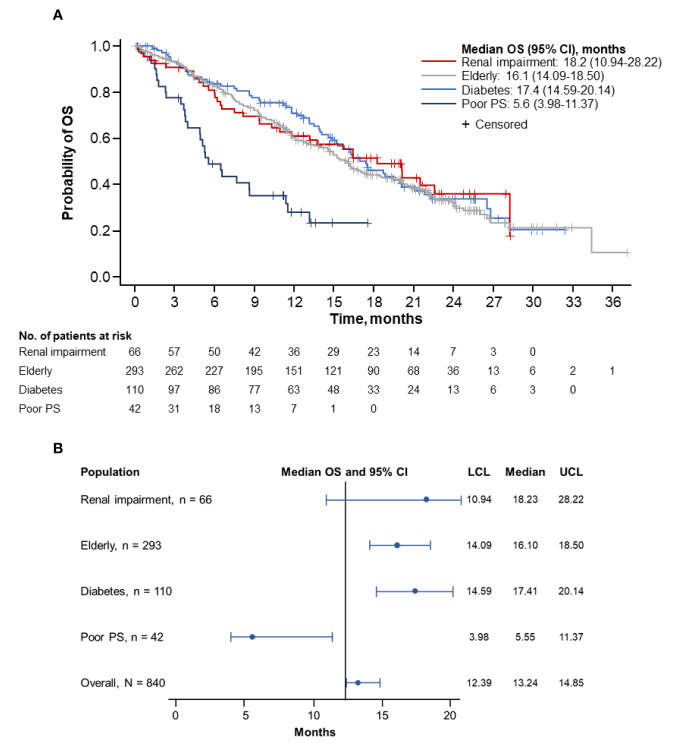
Kaplan-Meier curve **(A)** and forest plot **(B)** of OS by population. OS, overall survival; PS, performance status; LCL, lower confidence limit; UCL, upper confidence limit.

Overall, the longest median OS was observed in patients with renal impairment (18.2 months; 95% CI, 10.94–28.22 months), followed by patients with diabetes (17.4 months; 95% CI, 14.59–20.14 months) and by elderly patients (16.1 months; 95% CI, 14.09–18.50 months) (**Figures 2A, B****)**. Patients with ECOG PS 2 had the shortest median OS (5.6 months; 95% CI, 3.98–11.37 months). The corresponding 1-year OS rates were highest in patients with diabetes (71.1%; 95% CI, 61.11%–78.99%), followed by patients with renal impairment (61.3%; 95% CI, 47.95%–72.15%) and elderly patients (60.3%; 95% CI, 54.15%–65.84%). Patients with ECOG PS 2 had the lowest rate of survival at 1-year (28.4%; 95% CI, 14.73%–43.78%). The 2-year OS rates were highest in patients with renal impairment (36.2%; 95% CI, 22.00%–50.57%), followed by patients with diabetes (34.0%; 95% CI, 23.53%–44.66%) and elderly patients (31.8%; 95% CI, 25.40%–38.31%). No patients with an ECOG PS 2 survived to 2 years.

Further comparative survival analyses were performed on specific populations. The median OS was numerically longer in patients with (n = 66) vs without (n = 774) renal impairment (18.2 months [95% CI, 10.94–28.22 months] vs 13.0 months [95% CI, 12.22–14.55 months]; **Supplemental Figure 2A**). A longer median OS was observed in patients aged ≥ 70 years (n = 293) than in those under 70 years old (n = 547) (16.1 months [95% CI, 14.09–18.50 months] vs 12.4 months [95% CI, 11.17–13.73 months]; **Supplemental Figure 2B**). The median OS was also longer in patients with diabetes (n = 110) than in those without diabetes (n = 730) (17.4 months [95% CI, 14.59–20.14 months] vs 12.6 months [95% CI, 11.63–14.39 months]; **Supplemental Figure 2C**). However, the median OS was numerically shorter in patients with diabetes who received metformin (n = 59) (15.2 months [95% CI, 13.14–19.52 months]) vs those who did not (n = 51) (19.9 months [95% CI, 16.30–28.22 months]) (**Supplemental Figure 2D**).

#### Overall Response

**Figure 3:**
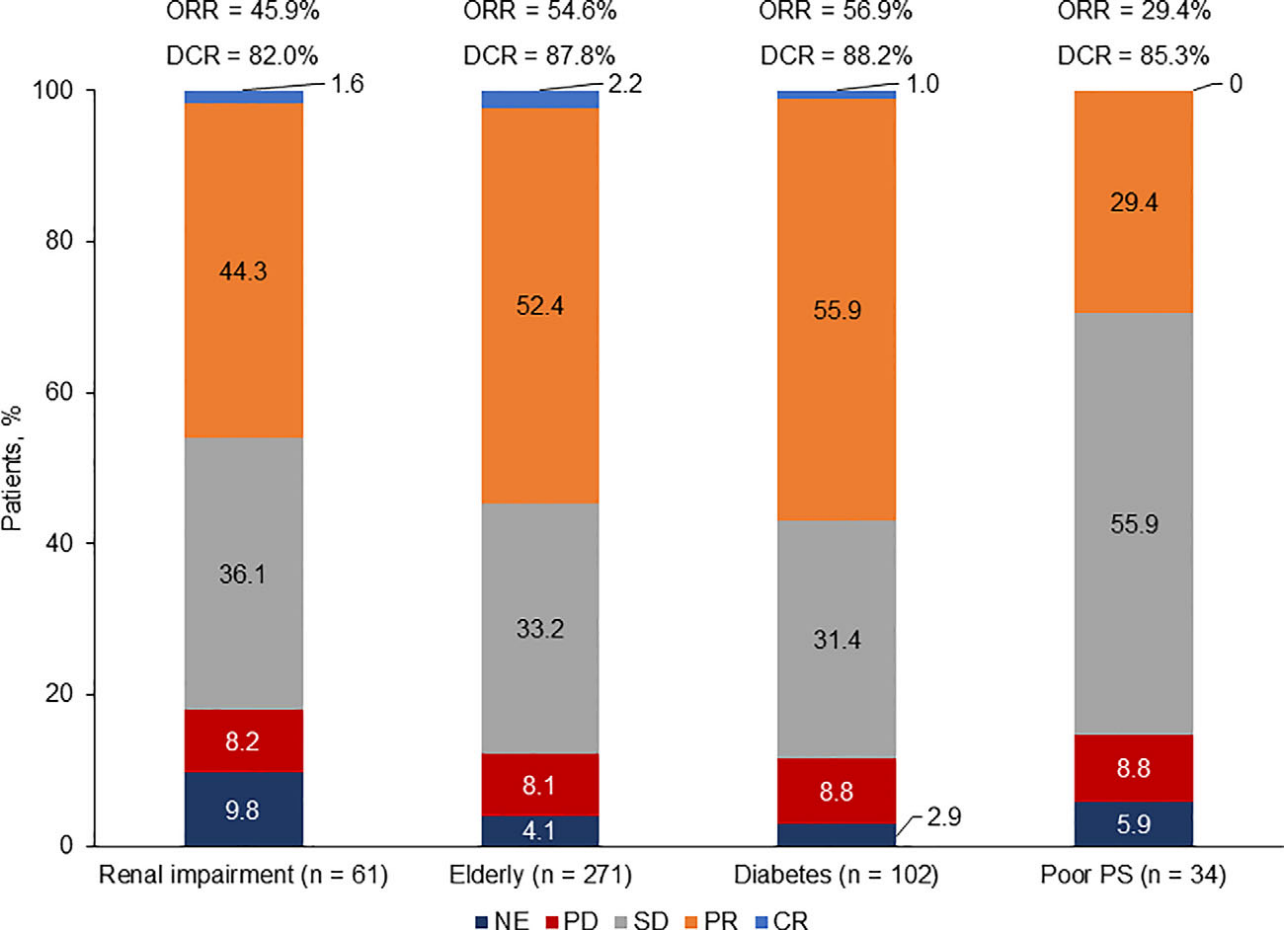
Response rate by population. CR, complete response; DCR, disease control rate; NE, not evaluable; ORR, overall response rate; PD, progressive disease; PR, partial response; PS, performance status; SD, stable disease. Based on patients with baseline and at least one post baseline tumor assessment.

Assessment of best overall response (by investigator in all studies except for the phase III trial by Socinski et al, which was performed by an independent radiology review committee) revealed that patients with diabetes exhibited the highest response rate [overall response rate (ORR): 56.9%; disease control rate (DCR): 88.2%], followed by elderly patients (ORR: 54.6%; DCR: 87.8%) and patients with renal impairment (ORR: 45.9%; DCR: 82.0%). Patients with ECOG PS 2 had the lowest response rate (ORR: 29.4%; DCR: 85.3%) (**Figure 3**).

### Treatment Exposure

Across populations, the median relative dose intensity (percentage of planned dose received) for *nab*-paclitaxel was 72.4% to 79.9% (**Table 2**). The median number of treatment cycles ranged from 4.0 to 6.0. Dose modifications, including reductions, interruptions, and delays, are described in **Table 2**.

**Table 2:** Treatment exposure and dose modification.

Parameter	Renal Impairment (n = 66)	Elderly (n = 287)	Diabetes (n = 107)	Poor PS (n = 42)
**Treatment Exposure**				
**No. of cycles, median (range)**	5.0 (1.0–26.0)	6.0 (1.0–48.0)	6.0 (1.0–34.0)	4.0 (1.0–18.0)
**Patients who received ≤ 6 cycles, n (%)**	48 (72.7)	198 (69.0)	64 (59.8)	32 (76.2)
**Dose intensity, median (range)**				
Carboplatin, mg•min/ml/wk	1.9 (0.2–201.7)	1.4 (0.1–226.9)	1.4 (0.3–255.6)	1.4 (0.4–226.0)
*nab*-Paclitaxel, mg/m^2^/wk	66.3 (26.7–102.4)	62.2 (14.5–116.7)	63.6 (14.5–100.8)	55.1 (31.4–100.0)
**Relative dose intensity, median (range), %**				
Carboplatin	87.6 (19.6–116.7)	82.5 (19.6–400.0)	83.2 (21.6–125.0)	83.8 (21.6–110.3)
*nab*-Paclitaxel	76.9 (26.7–102.4)	72.4 (19.3–155.6)	74.1 (19.3–105.2)	79.9 (47.1–100.0)
**Cumulative dose, median (range)**				
Carboplatin, mg•min/ml	30.0	24.0	24.0	19.5
	(5.0–14252.0)	(5.0–7974.0)	(5.0–9466.0)	(5.0–1840.0)
* nab*-Paclitaxel, mg/m^2^	1037.5	1200.0	1300.0	600.0
	(100.0–5150.0)	(100.0–9550.0)	(100.0–6675.0)	(100.0–3200.0)
**Dose Modification**				
**Patients with ≥ 1 dose reduction, n (%)**				
Carboplatin * nab*-Paclitaxel	43 (65.2) 43 (65.2)	189 (65.9) 200 (69.7)	65 (60.7) 69 (64.5)	13 (31.0) 15 (35.7)
**Patients with ≥ 1 dose interruption, n (%)**				
Carboplatin	0	0	2 (1.9)	0
* nab*-Paclitaxel	0	1 (0.3)	2 (1.9)	0
**Patients with ≥ 1 dose delay, n (%)**				
Carboplatin	55 (83.3)	215 (74.9)	79 (73.8)	16 (38.1)
*nab*-Paclitaxel	64 (97.0)	245 (85.4)	93 (86.9)	20 (47.6)

ECOG PS, Eastern Cooperative Oncology Group performance status.

### Safety

For patients with renal impairment, elderly patients, patients with diabetes, and patients with ECOG PS 2, treatment-related treatment emergent adverse event (TEAE) rates were 56.1%, 71.8%, 67.3%, and 85.7%, respectively; specifically for serious events, the rates were 10.6%, 13.2%, 13.1%, and 31.0%, respectively. The pooled analysis showed most treatment-related adverse events were hematologic in nature (**Table 3**). For patients with renal impairment, elderly patients, patients with diabetes, and patients with ECOG PS 2, grade 3/4 treatment-related hematologic adverse events included neutropenia (27.3%, 30.7%, 28.0%, and 16.7%, respectively), anemia (15.2%, 13.6%, 13.1%, and 16.7%, respectively), and thrombocytopenia (9.1%, 9.4%, 8.4%, and 4.8%, respectively). Additional adverse events of interest are shown in **Table 3**. Of note, grade 3/4 peripheral neuropathy was most common in patients with diabetes (18.7%), followed by elderly patients (12.9%), patients with renal impairment (7.6%), and patients with ECOG PS 2 (2.4%). Also noteworthy was a single occurrence of grade 3/4 acute kidney injury among patients with renal impairment. Grade 5 TEAEs were mainly cardiac in nature; elderly patients, patients with ECOG PS 2, and patients with diabetes experienced four (cardiac arrest, 2; cardiorespiratory arrest, 1; myocardial infarction, 1), three (cardiac arrest, 2; arrythmia, 1), and one (myocardial infarction) events, respectively. Additional grade 5 TEAEs were related to infections [two events in the elderly population (pneumonia, 1; sepsis, 1)], general disorders [one event each in the diabetes (disease progression) and elderly (death) populations], renal and urinary disorders [one event each (renal failure) in the renal impairment and elderly populations], and respiratory, thoracic, and mediastinal disorders [one event each in the renal impairment (pulmonary embolism) and elderly (acute respiratory failure) populations].

**Table 3:** Treatment-related TEAEs (≥ 5%) and other AEs of interest.

Grade 3/4 Adverse Event, n (%)	Renal Impairment(n = 66)	Elderly(n = 287)	Diabetes(n = 107)		Poor PS(n = 42)
**Treatment-Related TEAEs**					
Neutropenia	18 (27.3)	88 (30.7)		30 (28.0)	7 (16.7)
Anemia	10 (15.2)	39 (13.6)		14 (13.1)	7 (16.7)
Leukopenia	6 (9.1)	27 (9.4)		10 (9.3)	3 (7.1)
Thrombocytopenia	6 (9.1)	27 (9.4)		9 (8.4)	2 (4.8)
Fatigue	5 (7.6)	24 (8.4)		6 (5.6)	2 (4.8)
Neutrophil count decreased	1 (1.5)	20 (7.0)		2 (1.9)	0
Pneumonia	1 (1.5)	5 (1.7)		2 (1.9)	3 (7.1)
Asthenia	2 (3.0)	4 (1.4)		1 (0.9)	3 (7.1)
**AEs of Interest**					
Peripheral neuropathy^a,b^	5 (7.6)	37 (12.9)	20 (18.7)		1 (2.4)
Infections and infestations^a,c^	7 (10.6)	25 (8.7)	8 (7.5)		9 (21.4)
Febrile neutropenia	1 (1.5)	6 (2.1)	2 (1.9)		3 (7.1)
Myalgia and arthralgia^a,d^	0	2 (0.7)	1 (0.9)		0

AE, adverse event; PS, performance status; TEAE, treatment-emergent adverse event.

^a^Reported as a grouped term. ^b^Peripheral neuropathy grouped term includes peripheral sensory neuropathy, neuropathy peripheral, muscular weakness, peripheral motor neuropathy, paresthesia, gait disturbance, hypoesthesia, peripheral sensorimotor neuropathy, areflexia, burning sensation, peroneal nerve palsy, polyneuropathy, and muscle atrophy. ^c^Infections and infestations grouped term includes biliary sepsis, cellulitis, device-related infection, diarrhea infectious, diverticulitis, infective exacerbation of chronic obstructive airways disease, esophageal candidiasis, oral candidiasis, osteomyelitis, pneumonia, pneumonia streptococcal, sepsis, Serratia infection, urinary tract infection, and wound infection (2 elderly patients [0.7%] experienced grade 5 infections/infestations [pneumonia and sepsis, 1 patient each]). ^d^Myalgia and arthralgia grouped term includes arthralgia and myalgia.

## Discussion

Overall, the efficacy and safety results of this pooled analysis demonstrate that *nab*-paclitaxel–based regimens are reasonably well tolerated and may benefit patients with advanced NSCLC who are elderly (≥ 70 years) or have diabetes, renal impairment (eGFR < 60 ml/min/1.73 m^2^), or poor performance status (ECOG PS 2). Based on their efficacy and toxicity profiles, to date, *nab*-paclitaxel–based regimens are broadly applicable to the general NSCLC population and have been used as a platform for the development of new immunotherapy/chemotherapy combinations (18, 19). Patients with poor PS may fare worse, potentially due to premature treatment discontinuation (as supported by the higher rates of treatment discontinuation due to death, adverse events, or symptomatic deterioration in this population relative to the others in our analysis), but still stand to derive some benefit from this strategy.

Defining overall fitness of patients with advanced NSCLC and the influence of specific factors on suitability for chemotherapy, as they relate to types of chemotherapy or the option to withhold chemotherapy altogether, has been an ongoing area of interest for facilitating clinical practice decisions (20). Generally, there are few absolute restrictions that preclude chemotherapy (including those based on age or renal function), except for patients with poor PS, for whom the use of combination and in some cases single-agent regimens should be limited.

Carboplatin is an appropriate platinum backbone for patients with insufficient renal function, as cisplatin-based chemotherapy has been associated with severe nephrotoxicity as well as other toxicities including greater nausea and vomiting, ototoxicity, and neuropathy (21, 22). Further, *nab*-paclitaxel is predominantly eliminated *via* fecal rather than renal excretion (12). Collectively, these properties suggest that, when used together, *nab*-paclitaxel and carboplatin is a reasonable treatment option for patients with renal impairment. The current pooled analysis revealed 1 grade 3/4 acute kidney injury and 1 grade 5 renal event in patients with renal impairment. Further, patients with renal impairment unexpectedly demonstrated longer median OS than those without impairment (18.2 vs 13.0 months). This likely reflects the imbalance in patient numbers, which included only 66 patients with vs 774 patients without renal impairment, thereby resulting in a wide 95% CI for the renal impairment cohort and crossing of the survival curves.

In elderly patients, including those with ECOG PS 2, taxane-based chemotherapy doublets have demonstrated significantly longer PFS than vinorelbine or gemcitabine monotherapy (median PFS, 6.0 vs 2.8 months; *P* < 0.001) albeit with more frequent toxicity (23). Furthermore, the subgroup analysis of elderly patients enrolled in the pivotal phase III study demonstrated significantly longer median OS in those treated with *nab*-paclitaxel/carboplatin (19.9 months) compared with solvent-based paclitaxel/carboplatin (10.4 months; *P* = 0.009) (14, 17). The current analysis demonstrated numerically longer median OS in elderly patients than in patients < 70 years old, which suggests that *nab*-paclitaxel is a suitable combination partner in this older, vulnerable population. The data presented here, as well as data reported elsewhere, support the notion that cytotoxic chemotherapy doublets are efficacious and feasible for elderly patients.

The impact of concurrent diagnoses of diabetes and lung cancer on survival outcomes is variable (13). Several studies have demonstrated that patients with diabetes mellitus in addition to NSCLC experience a shorter OS than patients without diabetes (24–26). However, some evidence exists for prolonged survival in patients with diabetes compared with those without diabetes (27). In this pooled analysis, patients with diabetes exhibited longer median OS than those without diabetes (17.4 vs 12.6 months). The explanation for these results remains unknown.

Some studies have suggested an association between metformin treatment and improved outcomes in patients with diabetes and various solid tumors (27). In our pooled analysis presented, patients with diabetes who were treated with metformin had a shorter median OS than those who were not treated with metformin (15.2 vs 19.9 months). These results are consistent with the subgroup analysis of patients with NSCLC and diabetes by Hirsh et al. (13). Together, these results suggest that the hypothesized beneficial effect associated with metformin may not be applicable to *nab*-paclitaxel treatment in patients with diabetes.

A modest survival improvement in patients with NSCLC and ECOG PS 2 (including elderly patients) has been reported previously in those treated with combination chemotherapy vs single-agent regimens (median OS, 8.0 vs 6.6 months; *P* = 0.184) (28). Patients in the pooled analysis with ECOG PS 2 demonstrated a numerically lower median OS (5.6 months) than those in the Lilenbaum study. However, in the prospective ABOUND.PS2 study, median OS was 7.7 months (15). Patients with advanced NSCLC and ECOG PS 2 generally exhibit shorter OS than patients with ECOG PS 0 or 1. It is noteworthy that this subgroup of patients continues to demonstrate worse survival than the overall population, despite the use of checkpoint inhibitors (29). The presence of comorbidities poses a greater concern for these patients—for example, in the current analysis, 14% of patients with an ECOG PS 2 had moderate renal impairment at baseline, which is concerning given their age compared with other populations. Further studies are warranted to identify the reasons for such poor outcomes, which should include identification of predictive or prognostic biomarkers that may offer potential targets for therapeutic intervention.

The results from this pooled analysis contribute to our understanding of the role of combination chemotherapies in underrepresented patient populations, which historically have presented unique treatment challenges. Overarching themes spanning these populations include a lack of specific evidence from treatment experiences and the assumption that treatment may be associated with much worse toxicities due to comorbidities. The paucity of evidence has led to a lack of specific treatment recommendations in these patient populations. Thus, treatment decisions may be based on extrapolations from other trials or from prior experience. Perception of heightened toxicity may lead to undertreatment or, in some cases, to no treatment at all in patients who might otherwise benefit. The data presented here provide additional support for the role of *nab*-paclitaxel–based treatment regimens in elderly patients as well as patients with renal impairment, diabetes, and ECOG PS 2.

Our study has limitations, which must be taken into consideration when examining the data. For example, the pooled analysis vs a randomized clinical trial in a dedicated population does not account for differences in dosing schedules and treatment regimens between trials. In addition, some populations, such as those with ECOG PS 2, included a relatively small number of patients; this is not surprising, as most of the studies excluded patients with ECOG PS > 1. While our analysis was designed to focus on populations that tend to be poorly represented in clinical trials, it is possible that the resultant small sample sizes may have influenced the unexpectedly favorable survival results. Furthermore, the patients included in this pooled analysis were not restricted to a single analytic cohort; therefore, it is important to keep in mind when considering outcomes that patients may have had additional comorbidities beyond those highlighted in the specific comparison.

*nab*-Paclitaxel–based regimens are effective in populations frequently underrepresented in clinical trials, including elderly patients and patients with renal impairment, diabetes, and poor performance status. In addition, there does not appear to be an obvious exacerbation of toxicity in these populations. These results support the role of *nab*-paclitaxel–based chemotherapy regimens as a standard of care in a variety of patient populations, including those heretofore underserved in clinical trials, and make this regimen appealing as a platform for the development of immunotherapy.

## Data Availability Statement

The datasets presented in this article are not readily available because requestors must complete a data request on the Vivli platform. If the request is approved, Celgene will upload the anonymized data into the Vivli platform for use by researchers. Requests to access the datasets should be directed to https://vivli.org/ourmember/celgene/.

## Ethics Statement

All relevant ethical approvals from institutional review board/independent ethics committee have been obtained prior to study commencement. Written informed consent was obtained from all patients prior to study entry.

## Author Contributions

CJL, DM, JW, RB, MW, TJO, and MS: conceived of and designed the study. CJL, AG, CG, KK, DM, DS, DT, MT, JW, and MS: contributed to data collection. CJL, RP, RB, MW, TJO, and MS: analyzed the data. All authors: interpreted the data. All authors: revised the report critically. All authors contributed to the article and approved the submitted version. All authors agreed to be accountable for all aspects of the work and to ensure that questions related to the accuracy or integrity of any part of the work are appropriately investigated and resolved.

## Funding

Celgene, a wholly owned subsidiary of Bristol Myers Squibb, sponsored the study and was involved in the study design, data collection, and data analysis. All authors had full access to all collected data and had sole discretion in the data interpretation, writing of the report, and the decision to submit for publication. The corresponding authors had full access to all data in the study and had final responsibility for the decision to submit for publication.

## Conflict of Interest

CJL: Consultant/advisory fees, Celgene Corporation; other consulting fees: AstraZeneca, Bristol Myers Squibb, Genentech/Roche, Novartis, Pfizer, Takeda, Hospira, Merck, Boehringer Ingelheim. AG: Honoraria for Advisory Board, AstraZeneca; other fees, ICON Plc, CRO. CG: Advisory Board and Speakers’ Bureau member, MSD, Bristol Myers Squibb, Roche, AstraZeneca. DM: Advisory/Consultant, AbbVie, Bristol Myers Squibb, PharmaMar, Takeda. DS: Consulting or advisory role and research funding, AstraZeneca, Boehringer Ingelheim, Bristol Myers Squibb, Celgene Corporation, Genentech/Roche, Lilly, Novartis, Pfizer; research funding, Merck, University of Texas Southwestern Medical Center—Simmons Cancer Center. MT: Grants, Celgene Corporation, Bristol Myers Squibb, Roche, AstraZeneca; consulting fees, Celgene Corporation, AbbVie, Bristol Myers Squibb, Boehringer Ingelheim, Lilly, MSD, Novartis, Roche. JW: Grants, Celgene Corporation; consulting fees, Celgene Corporation. RP: Employment, Bristol Myers Squibb. RB: Employment, Bristol Myers Squibb. MW: Consulting fees, Bristol Myers Squibb. TJO: Employment, Bristol Myers Squibb.

The remaining authors declare that the research was conducted in the absence of any commercial or financial relationships that could be construed as a potential conflict of interest.

The authors declare that this study received funding from Celgene, a wholly owned subsidiary of Bristol Myers Squibb. The funder had the following involvement with the study: study design, data collection and data analysis.
