# Supplementary material for: nab-Paclitaxel/Carboplatin in Vulnerable Populations With Advanced Non-Small Cell Lung Cancer: Pooled Analysis
Source: Front Oncol. 2021 Jan 26;10:485587. doi: 10.3389/fonc.2020.485587 (PMC7871002; doi:10.3389/fonc.2020.485587)
Supplement: Supplementary file 1 [file DataSheet_1.docx]

*nab*-Paclitaxel/Carboplatin in Vulnerable Populations with Advanced Non-Small Cell Lung Cancer: Pooled Analysis

**Authors:** Corey J. Langer^1^, Ajeet Gajra^2^, Cesare Gridelli^3^, Kartik Konduri^4^, Daniel Morgensztern^5^, David Spigel^6^, Denis Talbot^7^, Michael Thomas^8^, Jared Weiss^9^, Richard Pilot^10^, Rafia Bhore^10^, Marianne Wolfsteiner^11^, Teng Jin Ong^10^, Mark Socinski^12^

Supplemental Materials

Supplemental Table 1. Pooled NSCLC studies

| Study Name | Study Number | Study Title | Number of Patients Pooled Into Analysis |
| --- | --- | --- | --- |
| Phase III trial (Socinski et al) | NCT00540514 | A randomized, phase III trial of *nab*-paclitaxel (ABI-007) and carboplatin compared with taxol and carboplatin as first-line therapy in patients with advanced NSCLC | 116 |
| ABOUND.SQM | NCT02027428 | A phase III, randomized, open-label, multicenter, safety and efficacy study to evaluate *nab*-paclitaxel as maintenance treatment after induction with *nab*-paclitaxel plus carboplatin in subjects with squamous cell NSCLC | 78 |
| ABOUND.PS2 | NCT02289456 | A phase II, single arm, open-label, multicenter, safety and tolerability trial with *nab*-paclitaxel plus carboplatin followed by *nab*-paclitaxel monotherapy as first-line treatment for subjects with locally advanced or metastatic NSCLC and an Eastern Cooperative Oncology Group performance status of 2 | 39 |
| ABOUND.70+ | NCT02151149 | Safety and efficacy of *nab*-paclitaxel in combination with carboplatin as first-line treatment in elderly subjects with advanced NSCLC: a phase IV, randomized, open-label, multicenter study | 143 |

Supplemental Table 2. Key inclusion and exclusion criteria of individual studies

| Study Name | Study Number | Key Inclusion | Key Exclusion |
| --- | --- | --- | --- |
| Phase III trial (Socinski et al) | NCT00540514 | - Histologically/cytologically confirmed nonresectable stage IIIB (with or without pleural effusion) or stage IV NSCLC measured by RECIST - ECOG PS 0 to 1 - Life expectancy >12 weeks - Previously untreated for metastatic disease - No radiotherapy within 4 weeks of enrollment - Prior adjuvant chemotherapy permitted if completed 12 months before study enrollment | - Untreated or symptomatic brain metastases - Neuropathy grade >1 - History of allergy or hypersensitivity to the study drugs |
| ABOUND.SQM | NCT02027428 | - Histologically or cytologically confirmed stage IIIB or IV squamous NSCLC measurable by RECIST version 1.1 - ECOG PS 0 or 1 - Previously untreated for metastatic disease | - Active brain metastases - Peripheral neuropathy grade ≥2 |
| ABOUND.PS2 | NCT02289456 | - Histologically or cytologically confirmed stage IIIB or IV NSCLC measured by RECIST version 1.1 - Not candidate for curative surgery or radiation therapy - ECOG PS 2 - Previously untreated for metastatic disease - Prior adjuvant chemotherapy permitted if completed 12 months before consent and without disease   recurrence) | - Active brain metastases - Peripheral neuropathy grade ≥2 |
| ABOUND.70+ | NCT02151149 | - Histologically or cytologically confirmed locally advanced or metastatic NSCLC measured by RECIST version 1.1 - ECOG PS 0 or 1 - Previously untreated for metastatic disease - If *EGFR* mutation or *ALK* gene translocation, must have had disease progression or proven intolerant to treatment with an EGFR or ALK inhibitor | - Active brain metastases - Peripheral neuropathy grade ≥2 |

ALK, anaplastic lymphoma kinase; ECOG PS, Eastern Cooperative Oncology Group performance status; EGFR, epithelial growth factor receptor; RECIST, Response Evaluation Criteria in Solid Tumors.

Supplemental Table 3. *nab*-Paclitaxel dosing

| Study Name | *nab*-Paclitaxel Treatment Arms to Pool |
| --- | --- |
| Phase III trial (Socinski et al) | Arm A   - *nab*-Paclitaxel 100 mg/m^2^ IV infusion over 30 minutes on days 1, 8, and 15 of each cycle - Carboplatin AUC = 6 mg•min/mL IV on day 1 of each cycle of each 21-day cycle after completion of *nab*-paclitaxel infusion |
| ABOUND.SQM | **Only patients who received induction and were randomized to receive *nab*-paclitaxel during maintenance were included in this pooled analysis**  Induction (4 cycles):   - *nab*-Paclitaxel 100 mg/m^2^ IV infusion over 30 minutes on days 1, 8, and 15 of each 21-day cycle - Carboplatin AUC = 6 mg•min/mL IV on day 1 of each 21-day cycle after completion of *nab*-paclitaxel infusion   Maintenance (until progression):   - *nab*-Paclitaxel 100 mg/m^2^ IV infusion over 30 minutes on days 1 and 8 of each 21-day cycle plus best supportive care until disease progression |
| ABOUND.PS2 | Induction (4 cycles):   - *nab*-Paclitaxel 100 mg/m^2^ IV infusion on days 1 and 8 of each 21-day cycle - Carboplatin AUC = 5 mg•min/mL IV on day 1 of each 21-day cycle after completion of *nab*-paclitaxel infusion   Maintenance (until progression):   - *nab*-Paclitaxel 100 mg/m^2^ IV infusion on days 1 and 8 of each 21-day cycle until disease progression or unacceptable toxicity |
| ABOUND.70+ | Treatment until progression or unacceptable toxicity  Arm A (21-day treatment cycle):   - *nab*-Paclitaxel 100 mg/m^2^ IV infusion over 30 minutes on days 1, 8, and 15 of each 21-day treatment cycle - Carboplatin AUC = 6 mg•min/mL IV on day 1 of each 21-day treatment cycle after completion of *nab*-paclitaxel infusion   Arm B (21-day treatment followed by 1-week break cycle; 28-day duration):   - *nab*-Paclitaxel 100 mg/m^2^ IV infusion over 30 minutes on days 1, 8, and 15 of each 21-day treatment followed by 1-week break cycle - Carboplatin AUC = 6 mg•min/mL IV on day 1 of each 21-day treatment followed by 1-week break cycle, after completion of *nab*-paclitaxel infusion |

Supplemental Figure 1. PFS Kaplan-Meier curves of populations of interest including patients with or without renal impairment (A), patients aged ≥ 70 or < 70 years (B), patients with or without diabetes (C), and patients with diabetes with or without metformin treatment (D)

A.


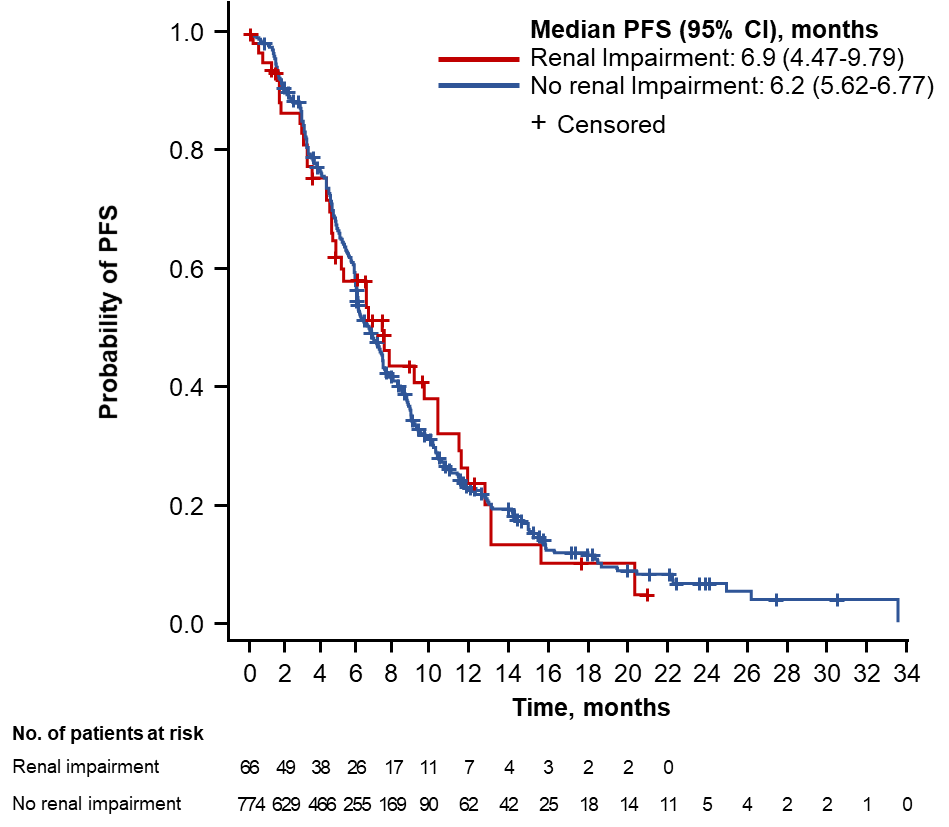


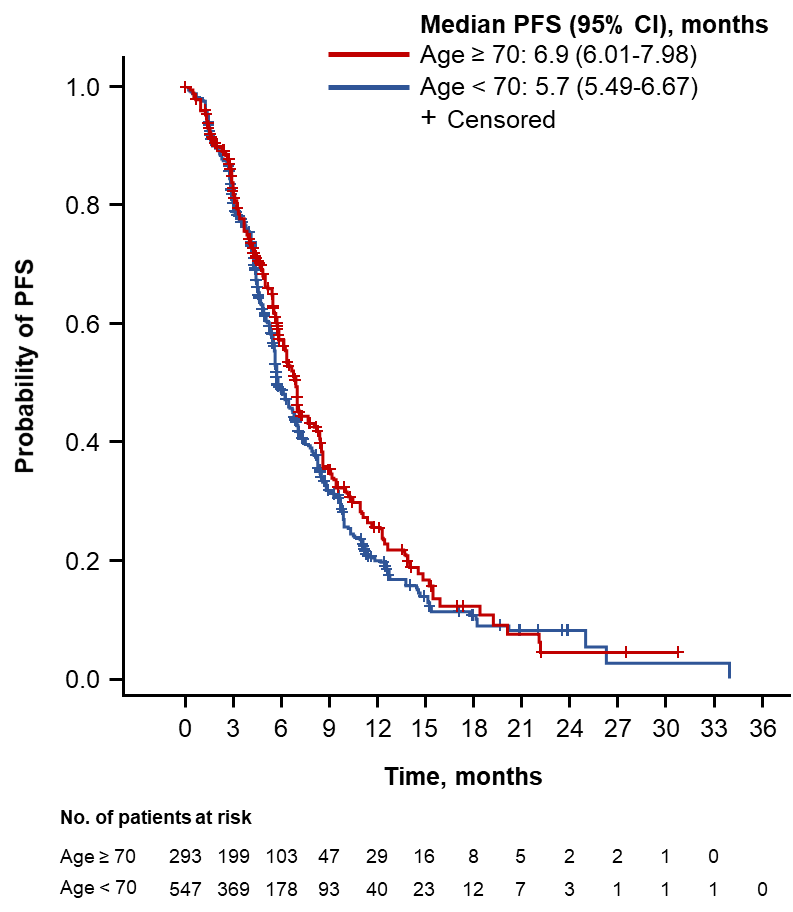
B.


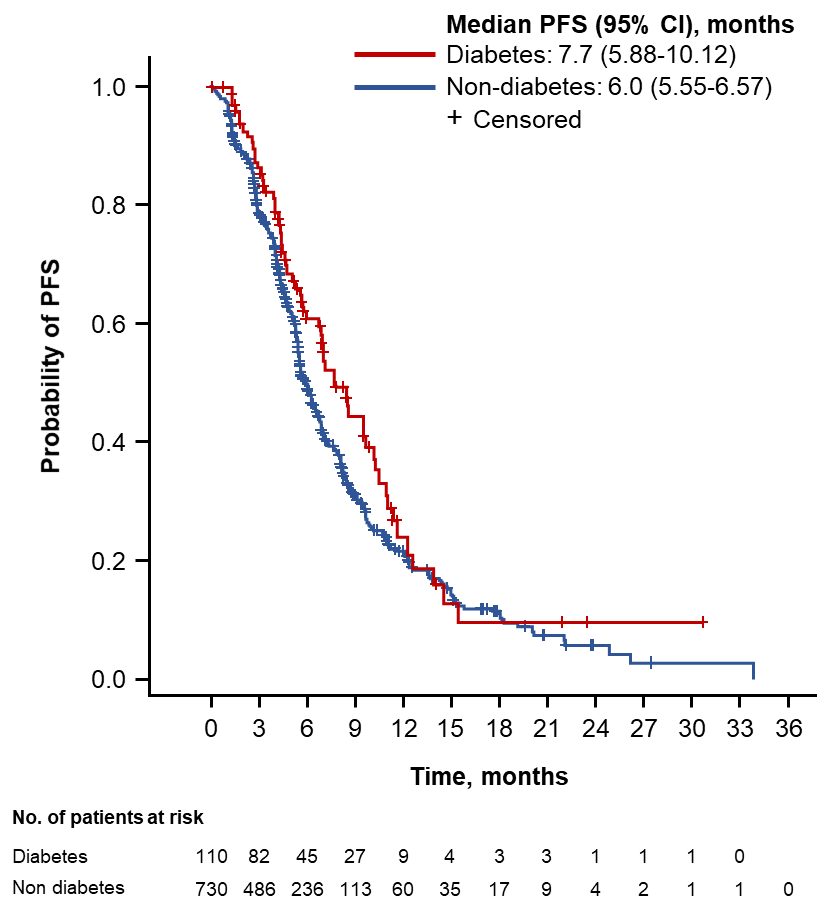
C.


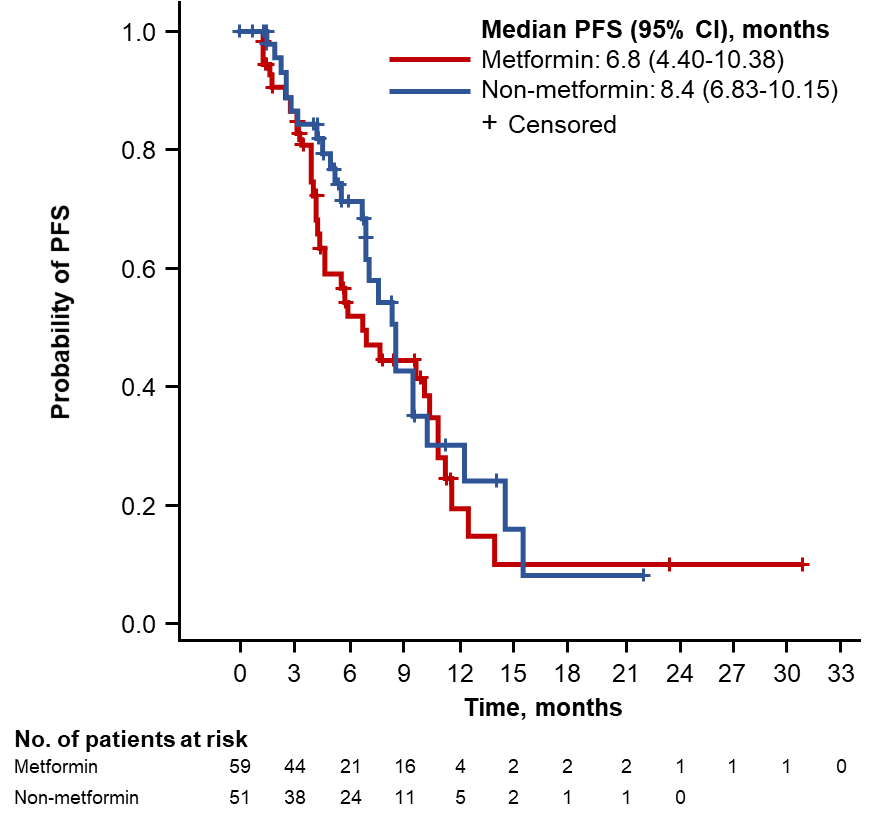
D.

Supplemental Figure 2. OS Kaplan-Meier curves of populations of interest including patients with or without renal impairment (A), patients aged ≥ 70 or < 70 years (B), patients with or without diabetes (C), and patients with diabetes with or without metformin treatment (D)

A.


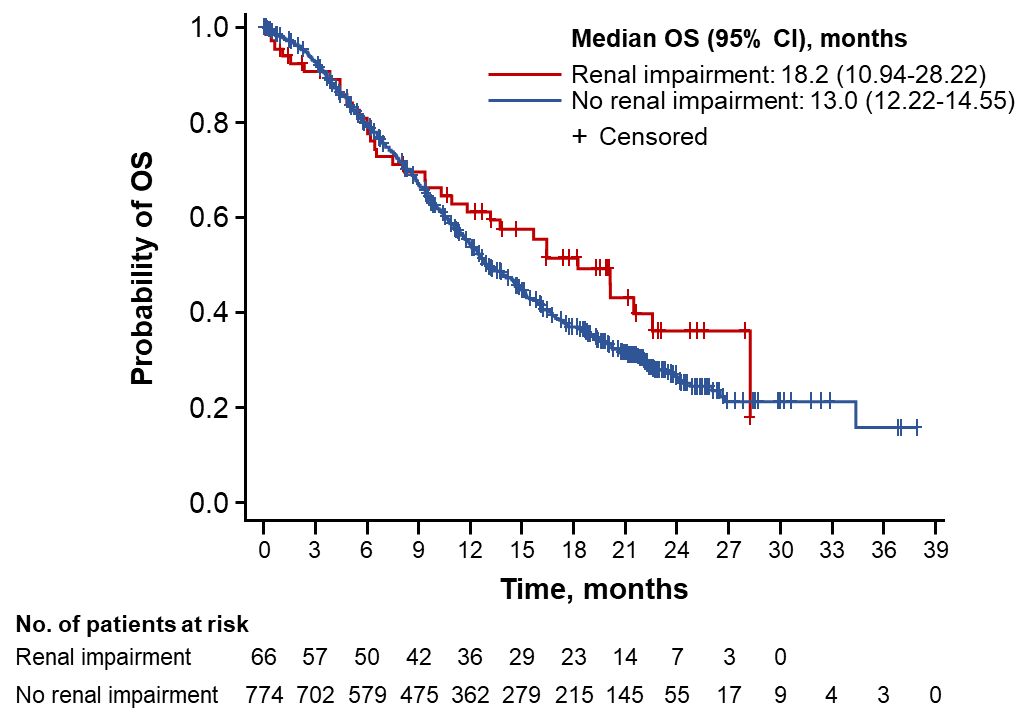


B.


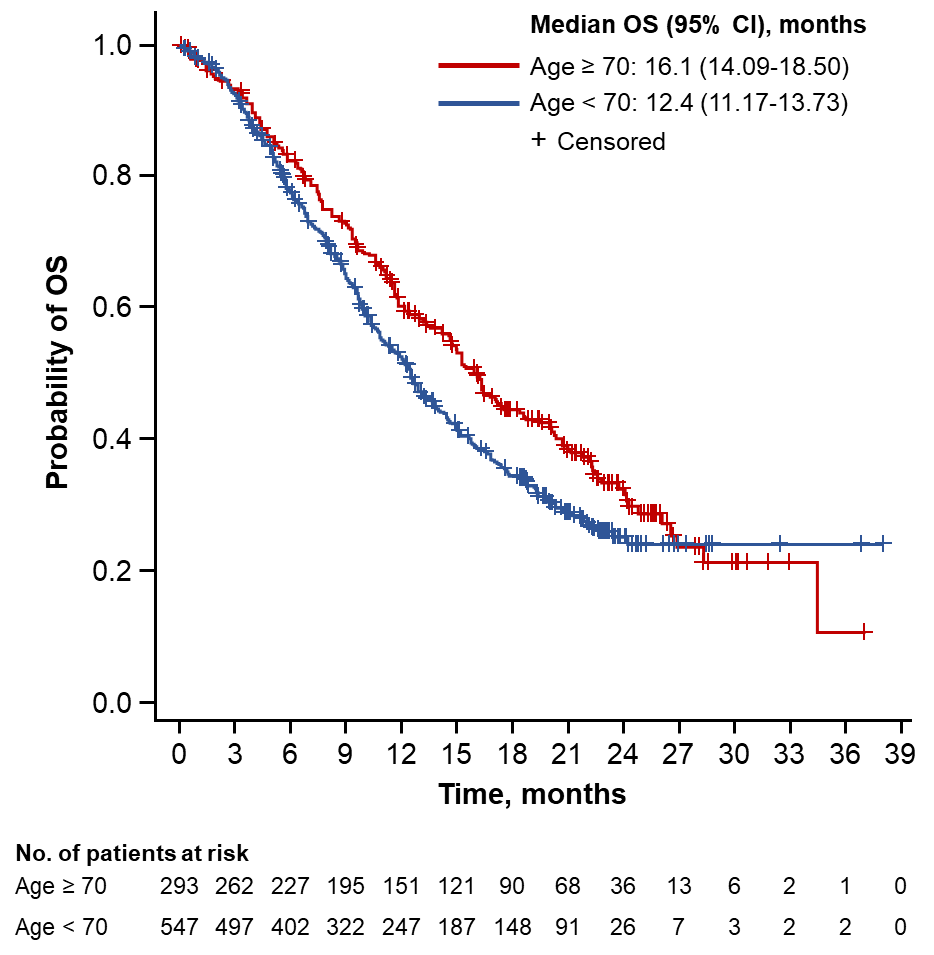


C.


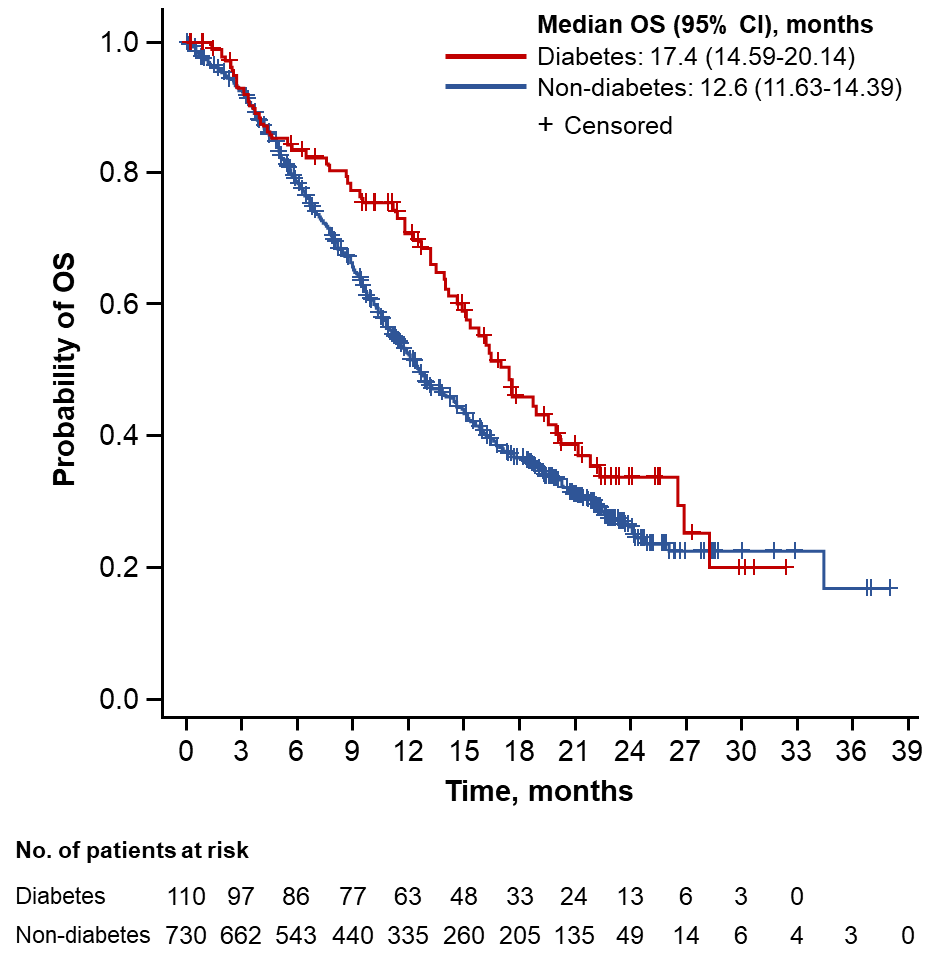


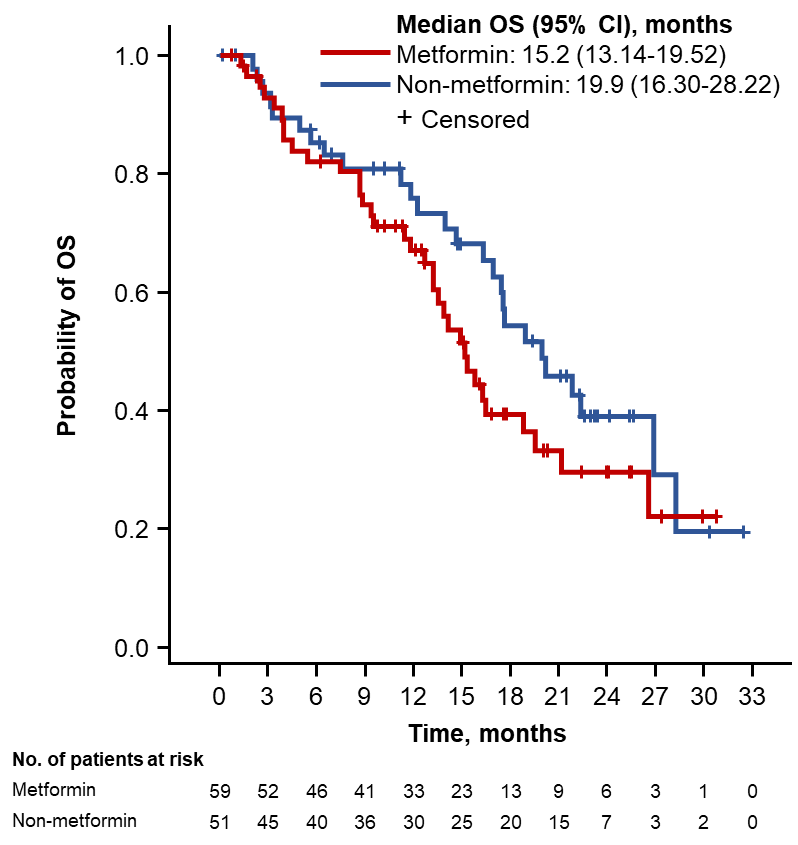
D.
